# Genetic Aspects of Dental Erosive Wear and Dental Caries

**DOI:** 10.1155/2021/5566733

**Published:** 2021-07-12

**Authors:** Amela Tulek, Aida Mulic, Maria Runningen, Jannike Lillemo, Tor Paaske Utheim, Qalbi Khan, Amer Sehic

**Affiliations:** ^1^Department of Oral Biology, Faculty of Dentistry, University of Oslo, Oslo, Norway; ^2^Nordic Institute of Dental Materials (NIOM), Oslo, Norway; ^3^Department of Maxillofacial Surgery, Oslo University Hospital, Ullevaal, Oslo, Norway; ^4^Department of Medical Biology, Faculty of Health Sciences, University of Tromsø—The Arctic University of Norway, Tromsø, Norway

## Abstract

**Objectives:**

The present review aims to give an overview of the literature focusing on novel genetic aspects of dental erosion and dental caries. Once the tooth erupts into the oral cavity, the regenerative capability of enamel is fundamentally limited due to the loss of dental epithelium during eruption. The susceptibility or resistance to dental erosion and caries is presumably a result of environmental, phenotypic, and/or genetic influence. Even though it is evident that individuals frequently exposing their teeth to acid and sugar are at high risk of developing dental erosion and caries, the findings exclusively based on these factors are elusive. *Data resources and study selection*. The present review was based on data collected from the National Library of Medicine database with different combinations of the following terms: “tooth,” “dental,” “dentin,” “enamel,” “erosion,” “erosive wear,” “caries,” “decay,” “gene,” and “genetic.” A total of forty-six studies met the inclusion criteria. Data were extracted by one reviewer and verified by another.

**Conclusion:**

The high prevalence of erosion and caries among certain groups, and observations that not all individuals appearing to be at risk develop these lesions, has sparked research on identifying genetic effects to these conditions. A connection of genome-wide and candidate gene studies has increased considerably in the literature. This review reveals largely varying success among studies, demonstrating the difficulties of developing the study with adequate sample sizes and durable phenotype definitions that permit enough statistical power to identify genetic contributors.

## 1. Etiology of Dental Erosion and Dental Caries

Mechanical and chemical processes, influencing each other, result in wear of hard dental substance. Dental erosion is a chemical, irreversible loss of dental hard tissue by acid and/or a chelator without bacterial involvement [1]. However, as teeth are rather exposed to combination of various types of wear such as attrition (tooth-tooth contact wear), abrasion (teeth to foreign substance contact wear), and abfraction (fatigue of the cervical part of the tooth), in addition to erosion, it is difficult to isolate these processes in one mouth. Therefore, the terms dental erosive wear and erosive tooth wear are also used to refer to the same phenomenon [2].

As many factors are associated with the pathology of dental erosion, it is commonly accepted to be a multifactorial condition caused by various extrinsic and intrinsic acid sources [3–6]. Extrinsic dental erosion can be a consequence of exposure to acid from industrial or dietary sources. These acids are mainly found in some types of food and drinks such as citrus fruits, acid beverages, and sour candies. Furthermore, some medications are associated with erosion, particularly antidepressants, asthma drug therapy, and chewable vitamin C tablets [7, 8]. Eating and drinking habits with a frequently intake of dietary acids, occupation, and socioeconomic aspects are also risk factors for dental erosion. Intrinsic dental erosion is generally caused by the exposure of teeth to stomach acid. This mainly occurs with frequent vomiting, in patients with regurgitations, and gastroesophageal reflux or ruminations [3, 5, 6, 9, 10]. Dental erosion has been described as a surface phenomenon, namely destruction that primarily affects the surface of the teeth. In addition, studies have shown that chemical attack also leads to demineralization just beneath the tooth surface. Therefore, it is also described as near-surface phenomenon [11]. Even though dental erosion appears in low pH, there is no fixed critical pH value for acidic solutions. The concentrations of calcium and phosphate ions in the erosive solution itself calculate the critical pH value for enamel. This differs from caries where the critical pH calculates from calcium and phosphate concentrations of plaque fluid. Furthermore, dental pellicle, type of organic acid, the acids' undissociated form, organic dentine matrix, and many other factors are involved in erosive tooth wear. Depending on the balance between patient-related and nutritional factors that interact with the tooth surface, the result will either be a protection or demineralization of the tooth surface. Therefore, it may be speculated that some individuals are more vulnerable for developing dental erosion than others. Moreover, although different patients are exposed to exactly the same acid in their diet, individual biological factors will also affect the risk of evolving erosive lesions [12].

Dental caries is a multifactorial and complex disease, which is the most prevalent disease in developing countries [13]. This is a chronic disease that can cause pain, suffering, and diminished quality of life, including eating and speaking [14]. The tooth surface is covered by dental plaque, which is a sticky biofilm constituted of diversity of bacterial species embedded in a polymer matrix [15]. A caries lesion is a result of a metabolic event in the dental plaque in which certain acidogenic bacteria cover the affected area, produce acid from fermentable carbohydrates, and lead to a localized chemical dissolution of the tooth. The caries process starts when there is an imbalance in the equilibrium between the tooth minerals and the dental plaque (biofilm). The enamel loses calcium and phosphate during acid exposure and contact with dental plaque. The result is a porous and visible enamel, which is called a “white spot” lesion [16, 17]. Like dental erosion, the caries process is a balance between pathological factors and protective factors. Caries lesions progress when the pathological factors outweigh the protective factors [18]. Cariogenic bacteria, fermentable carbohydrates, and salivary dysfunction are pathological factors leading to demineralization [19]. Regarding the tooth morphology, deep grooves and areas of retention are common locations for caries lesions. If the demineralization process is not halted or reversed, it will eventually lead to a cavity [20]. Oral hygiene, dietary choices, daily oral hygiene, and saliva composition and flow have an important influence on caries progression [16]. On the other hand, the frequency of food-intake and type of diet is also decisive for the development of caries. Together with dietary advice, the flow of saliva helps dislodge food and pathogens from the tooth surface almost immediately. Microbes clump together with saliva and have a shot time on the teeth [19].

It is worth to mention the adverse effect of orthodontic treatment as a risk factor for the development of erosive and carious lesions. Demineralization of the enamel surface, mostly due to inappropriate oral hygiene, has been noted in approximately 50% of orthodontic patients [21]. Furthermore, the vitality of pulp, soft dental tissue, and volume of the pulp chamber may be altered in orthodontic treatment. A recent study by Lo Giudice et al. has proposed a new 3D imaging technology that can detect morphological changes of dental hard and soft tissues in patients with orthodontic appliances [22]. Moreover, these individuals should be treated as a risk group and extensive advice on diet, oral hygiene techniques, and preventive regimes during the orthodontic therapy should be provided to them.

The prevalence and incidence of both caries and dental erosion is high, nowadays [14, 23]. Even though the pathological mechanisms of these two diseases are different, they share some common biological factors such as salivary components and flow rate, tooth formation and structure, immune response, or an individual's variation in taste preferences [24–26]. Additionally, these factors are under genetic control, and therefore, the genetic background features the dynamic of the development of oral diseases. The studies have shown that susceptibility to erosion and caries varies considerably among individuals exposed to similar risk [27–30]. It is plausible that genes regulate the structure of hard dental substance, the salivary composition and flow, the behavioral patterns, and the immune response. While traditional dentistry has put more focus on environmental risk factors for erosion and caries development, understanding the complex gene-environment relations opens doors for further associations yet to be discovered.

The aim of this study is to give an overview of the latest and most relevant data related to genetic aspects of dental erosion and dental caries.

## 2. Data resources and Study Selection

**Figure 1:**
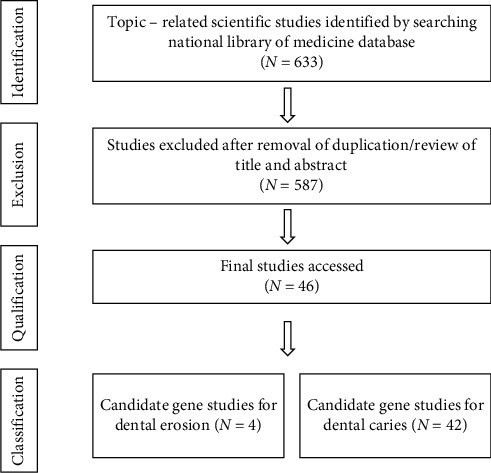
Studies selection protocol.

The present review was based on data collected from the National Library of Medicine database with different combinations of the following terms: (“tooth” OR “dental” OR “dentin” OR “enamel”) AND (“erosion” OR “erosive wear”) AND (“caries” OR “decay”) AND (“gene” OR “genetic”) in an attempt to reveal relevant publications. The search was performed without restriction on the study design or language. Selection criteria included articles published from 2005 to the present year that describe the pathogenesis, etiology, and other characteristics associated with the development of dental erosion and caries in humans. Primary search yielded 633 studies. All authors independently reviewed the titles and abstracts of the selected articles, applying the selection criteria mentioned above and excluding eventual duplicates. Articles were also rejected if they were clearly unqualified. In case where updated versions of the paper was found, older versions were rejected. The authors then reviewed the remaining articles to determine their eligibility. Finally, forty-six studies were accessed, out of which four studies were related to dental erosion and genetics and forty-two studies were related to dental caries and genetics. Summary of protocol for studies selection and research articles are presented in Figure 1 and Tables 1 and 2, respectively.

## 3. Genetic Control of Dental Enamel Formation

Ameloblasts, the enamel-forming cells that fabricate dental enamel, exhibit an incredible resistance to wear and fracture. Production of dental enamel, amelogenesis, can be divided into three stages of the ameloblast life cycle, i.e., secretory, transition, and maturation. The secretory stage ameloblasts secrete enamel matrix proteins until the full thickness of enamel is fulfilled, whereas the transition and maturation ameloblasts control the terminating mineralization of enamel in which the enamel matrix hardens as the hydroxyapatite crystallites increase growth in thickness and width [93]. Furthermore, the dental enamel formation is under specific and well-regulated genetic control [48]. Amelogenin (AMELX), ameloblastin (AMBN), and enamelin (ENAM) are structural, specific proteins of the dental enamel matrix [93]. Therefore, enamel malformation may be a result of defects in genes encoding enamel proteins [48]. Amelogenin (about 90% of the total enamel proteins), ameloblastin (5–10%), and enamelin (1–5%) are three major structural proteins [48]. These proteins are the most important secreted products of the secretory-stage ameloblasts, and their main functions are the organization of the enamel structure and increase of the length of existing crystallites [48, 93]. Amelogenin is the most abundant enamel protein. It is expressed from genes on the X- and Y-chromosomes and is considered as the most critical component in the matrix. Mutations that knock out the copy of human X-chromosomal amelogenin gene result in enamel malformation with both hypoplastic and/or hypomineralization defects [48]. Ameloblastin is a glycosylated protein. A short time after secretion, ameloblastin is proteolytically cleaved in its N-terminal half. The rest of the protein accumulates in the sheath space throughout the entire thickness of developing enamel. The proteolytic product can be detected only in the rod and interrod enamel within thirty micrometers of the enamel surface [48]. The largest enamel protein is enamelin. Approximately one-third of the molecular weight results from glycosylations. Enamelin is directly involved in the catalysis of crystal elongation and confined in its distribution of the mineralization. C-terminus of the enamelin is cleaved proteolytically, and its cleavage products are unstable and do not accumulate in the enamel matrix. The products that accumulate are localized to the prism and interprism enamel [48].

## 4. The Role of Saliva

Saliva is a crucial factor in maintaining the oral health. It is important for integrity of the dental enamel, dental caries, and dental erosion susceptibility. The characteristics of saliva are controlled by several factors, including genes [26, 48, 93]. Saliva has a protective and stability role in the enamel formation and structure. Dental enamel contains the highest proportions of minerals in the human body and is the hardest “tissue.” These minerals are calcium-deficient carbonated hydroxyapatite containing fluoride [94]. The mineralization of the dental enamel is mediated by the ameloblasts, several key genes involved in the formation, and the passage of mineral across cell and fluid barriers [26, 95]. It is beneficial that saliva is supersaturated with respect to enamel apatite. This enamel apatite will dissolve from the tooth if the level of calcium, phosphate, and fluoride ions in saliva decreases [96]. Salivary flow and components, its' antibacterial properties, and fluoride from extrinsic sources are protective factors and lead to remineralization [18]. On the other hand, polypharmacy, Sjögren's syndrome, and radiotherapy of head and neck cancer may influence the function of salivary glands leading to reduced saliva flow rates and abnormal saliva composition, which in turn may increase the predisposition to dental erosion and dental caries [97]. Kuchler et al. evaluated genetic variations in genes expressed in enamel development and their association with calcium and phosphorous levels in saliva. The study concluded that altered levels of calcium and phosphorous in saliva depends on genes coding for enamel matrix proteins during enamel development. Their results demonstrated that calcium levels were associated with genetic variation in AMELX, AMBN, and ESRRB [26]. Another important aspect of saliva is the salivary flow rate. An association between dental caries and amount of secreted saliva has been shown previously [98]. Salivary production and flow rate are to some extent determined by the function of aquaporins, a family of water channel proteins located in salivary glands [99]. A recent study found a positive association between severe erosive tooth wear and certain salivary aquaporin markers when using the logistic regression analysis. [52] (Table 1).

## 5. Current Treatment Modalities of Dental Erosion and Caries

Considering both short and long perspective, it can be provocative for the dentist to choose the most favorable treatment for each patient since there is no standard treatment recommended. Therefore, a minimal invasive conduct is the best option in most cases [23]. The treatment decisions of teeth affected by dental erosion among Nordic dentists have been evaluated [100–102]. Identifying, limiting, or eliminating the acid exposures are important aims for the dentist and the patient. Preventive treatment is to prefer before restorations and should always be recommended. For some individuals, the operative treatment may be required as additional treatment. In these cases, dependently on localization end extension of lesions, fillings with resin composite or glass ionomer cements are the first materials of choice before restorations with ceramic veneers, inlays, onlays, or crowns. Preventive treatment should include (1) information about good dietary and drinking habits, (2) rinsing with fluoride or use of fluoride tablets, (3) brushing technique and habits, and (4) referral to specialist faculty clinics or another dentist [23, 100]. Operative treatment options include local treatment with fluoride solution or bonding material to prevent dentin hypersensitivity, filling restoration with resin composite, restore with ceramic facet/inlay/onlay, and restore with crown [100, 103]. It is important for the dentist to have increased knowledge about eating disorders and its influence on oral health. This can help with early detection and intervention of dental erosions in patients suffering from eating disorders [104, 105]. Saliva stimulants may prevent development of dental erosion for irritated dry mouth patients with reduced salivary flow rate and/or with impaired saliva composition. Modifications with calcium will make acidic saliva stimulants nonerosive in healthy and dry mouth patients [106]. Therefore, health professionals should recommend saliva stimulants with significantly erosive potential for dry mouth relief [107–111].

Strategies to prevent dental caries development are either to attack forces that can be attenuated, or the host resistance can be fortified. The attacking forces decrease when dental plaque is regularly removed and consequently less acid is produced from metabolized dietary sugars. On the other hand, the host resistance can be enhanced by increasing the potential for remineralization of demineralized enamel with different fluoride-containing products [18]. Treatment modalities of dental caries are to arrest or reverse the progression. Featherstone described two bottom line principles: reduce the pathological factors and increase the protective factors [18]. Operative treatment options are current when destruction of the tooth is comprehensive and the remineralization as an option is not possible.

## 6. Genetic Contribution to Development of Dental Erosion and Caries

The present review was based on data collected from the National Library of Medicine database with the different combinations of the following terms: “tooth,” “dental,” “dentin,” “enamel,” “erosion,” “erosive wear,” “caries,” “decay,” “gene,” and “genetic,” in an attempt to reveal relevant publications. The search was performed without any restriction on the study design or language. However, after detailed analysis of all publications, a total of forty-six studies related directly to the core topic of the present review were used. A protocol for studies selection is presented in Figure 1. Studies and candidate genes for erosion and caries in humans may be found in Tables 1 and 2, respectively.

Despite frequent exposure of the tooth to acids and great risk of developing erosive wear on the tooth, several studies have shown that some individuals do not evolve erosive lesions [27, 112]. The interplay between high risk and rapid exposure to acid is considerable when endeavoring to explain why some tend to develop more or less erosive wear than others, regardless of exposure to the same acid and intensity. By now, there is no manifest association explaining why some individuals at risk have reduced susceptibility to erosive lesions. Furthermore, research and several studies have shown a higher prevalence among males than among females [28]. Acidic tooth wear, dental caries, diabetes, and heart disease may be referred to as complex disorders as they have both genetic and environmental influences. The analyses of the role of genetics when it comes to dental erosion may be complicated because of the significant environmental and behavioral contributions to the incidence of the disease [31]. Genome-wide association studies (GWAS) and candidate gene studies are two common ways to study the genetics of a complex trait. A GWAS is serving as a hypothesis-generating procedure as it investigates the linkage or associations between anonymous DNA variants with known locations throughout the genome. The candidate gene studies test hypotheses with regard to possible coherences between specific genes or gene variants and a disease or trait. Investigating single nucleotide polymorphisms (SNPs) in specific genes is used to detect genetic factors contributing to caries and how their assumed or known function is relevant to the disease. The majority of the data obtained from the genetic studies of dental erosion comes from the last decade. Enamel formation genes, immune response genes, genes related to saliva, and genes related to taste are some of the major candidate genes studied to date, of which the enamel formation genes are the most studied group [16]. It is reasonable to assume that variations in enamel formation genes may be involved in the susceptibility to dental erosions as several studies have shown positive associations between caries experience and variation in enamel formation genes. A clinical study by Sovik et al. found that there is an association between severity of dental erosions and genetic variation in enamel formation genes [31]. Nevertheless, it is important to remember that levels and frequency of acid exposure are hard to determine and control. Genetic correlations can be mimicked by investigating covariance between relatives with shared behavior, practice, and habits [31, 113]. Several studies have found that there are strong associations between dental erosion and genes coding for enamel matrix proteins (Table 1). A statistically significant association between tooth erosion and expression of enamelin (ENAM rs12640848) has been reported [31]. Furthermore, a clear connection between severe dental erosion and amelogenin expression (AMELX) rs946252 has been suggested (Table 1). Enamelin and amelogenin are both enamel formation genes involved in mineralization of the enamel. Uhlen et al. found no evidence of association between several markers of tuftelin 1 (TUFT1) and dental erosion. When analyzing the terciles, significant associations were found between enamel loss and the TUFT1 rs4970957 marker, tuftelin-interacting protein 11 (TFIP11) rs134136, TFIP11 rs134136, and AMELX rs946252. When comparing allele and genotype distributions between individuals more and less susceptible to enamel loss, no statistically significant differences were found [28]. Alaraudanjoki et al. found significant associations between dental erosion and PXDN, MYT1L, IRX1, IRX4 rs493321, and PRMT8 rs768398. When it comes to males, there was a significant association between dental wear and C9orf86, FGFR1 rs11993596, rs112007639, rs12546327, rs2461333, CDH4 rs2426986, rs16984837, rs6101273, and *γ*-protocadherin (PCDHG) chr5: 141405828. Among females, there was a significant association between dental wear and SCD5 chr4: 82684912, F2R, and F2RL1 chr5: 76795786 [34]. According to Uhlen et al., there is no evidence of association between dental erosion and ameloblastin (AMBN) rs4694075, tuftelin 1 (TUFT1) rs7526319, and tuftelin 1 (TUFT1) rs7526319 [28]. Another interesting gene is msh homeobox 1 (MSX1), which is a part of a large family of homeobox genes. It is involved in tooth development [50]. A study from 2019 found a cluster of suggestive SNPs near this gene to be associated with dental erosions [34]. However, these findings still require further research.

Several studies have suggested genetic factors affecting dental caries risk (Table 2). The genes of interest are mostly related to enamel formation, saliva and immune system, taste, and dietary habits. Enamel formation genes such as tuftelin, enamelin, amelogenin, and ameloblastin are associated with higher caries experiences (Table 2). Tuftelin and amelogenin are expressed in the developing and mineralized tooth. Enamelin controls the mineralization and structural organization of the enamel, whereas ameloblastin is involved in the enamel matrix formation and mineralization. Enamelin and amelogenin are mainly associated with high caries experience, even though some studies found no association. Two studies have shown that tuftelin interaction protein 11 also is associated with initiation of carious lesions and higher caries experiences. However, there are five studies showing no evidence of association. Kallikrein-related peptidase 4 (KLK4) and matrix metalloproteinases 20 and 16 (MMP20, MMP16) degrade amelogenin. KLK4 gene variations are associated with both lower and higher caries experiences, while MMP20 and MMP16 are associated with higher caries experiences combined with poor oral health. Additionally, matrix metalloproteinases 2, 9, and 13 have also been investigated in correlation with caries susceptibility (Table 2). Immune response genes have also been investigated. Results from several studies have shown that there is an association between immune response genes and caries risk. CD14 molecules mediate innate immune response to bacterial lipopolysaccharide, and they are linked to absent in saliva of individuals with active carious lesions. Human leukocyte antigen, a major histocompatibility complex class II, DR beta 1 (HLA-DRB1) and DQ beta 1 (HLA-DQB1) present peptides derived from extracellular protein. The frequency of allele 4 of DRB1 is increased in children with early childhood caries. Moreover, allele 2 of DQb1 is increased in adolescents affected by caries. DRB1 allele 1 and DQB1 allele 3 frequencies are increased in the presence of *Streptococcus mutans*. Beta defensin 1 (DEFB1) is an antimicrobial peptide implicated in the resistance of epithelial surface to microbial colonization, and it is associated with low and high caries experiences. The immune gene lactotransferrin (LTF) is an iron-binding protein in milk and body secretions with antimicrobial activity and is not linked to high risk of caries. Mucin 7 (MUC7) facilitates the clearance of bacteria in the oral cavity, and absent is associated with higher caries experience. Mannose-binding lections (protein C) 2, soluble MBL2 have no evidence of association. Absence of aquaporin 5 (AQP5) is associated with higher caries experience. Protein-rich protein HaeIII subfamily 1 (PRH) provides a protective environment for the teeth, and absent is associated with higher caries experiences and colonization by *Streptococcus mutans*. A recent study found associations between carbonic anhydrase VI (CA6), *S. mutans* colonization, tooth biofilm microbiota composition, and risk of dental caries. Carbonic anhydrase VI is suggested to be involved in saliva formation, regulation of pH, and tooth formation. Finally, taste preference is a result of genetic and environment influences. In accordance with this, some studies have found the association of different caries severity and variations in taste and dietary preference genes, TAS2R38, TAS1R2, and GNAT3. A detailed overview is presented in Table 2.

## 7. Conclusion and Future Perspectives

The susceptibility to dental erosion and caries varies between individuals and seems to be affected by oral environment and variations in the dental enamel. Several studies have demonstrated an association between dental caries, dental erosive wear, and genetic factors. The genes coding for enamel matrix proteins, i.e., amelogenin, enamelin, tuftelin, and tuftelin interaction protein 11, are associated with increased susceptibility to both dental erosion and caries. Enormous progress and interest in identifying the genetic factors influencing the development of erosion and caries is evident. Both diseases are still highly prevalent worldwide, and it has been more than a century of investigation of their pathogenesis. New strategies that can protect individuals at higher risk are warranted. Recently, studies have identified promising biomarkers that aim to help in personalizing treatment for individuals at higher risk to develop dental erosion and dental caries. Even though genetic studies aim to investigate the underlying susceptibility and reveal the risk prior to the disease/condition onset, follow-up studies are needed to replicate the initial findings. Challenges can be met when determining the presence or the level of the disease. For that reason, studies of the same associations can have inconsistent results. Furthermore, in population-based case-control studies, the associations can be confused by differences in the sex ratio between cases and controls. Additionally, the occurrence of X inactivation, affecting random loci on the X chromosome in females, results in different risk between males and females. Future studies should focus on phenotypic definitions and include demographic variables and environmental exposures in their analysis. Clinical studies such as randomized trials may also result in novel insights. Collectively, a combination of genetic analyses such as gene expression, metagenomics, and protein-protein interaction networks may increase our knowledge and help us understand dental erosion and caries. Moreover, comprehensive information about hereditary contributor could help identify patients at risk prior to its occurrence. Designing a prognostic genetic test with a predictive ability may pave the way for developing more targeted therapies that precisely address the personal risk.

## Figures and Tables

**Table 1:** Candidate genes studied for erosion in humans.

Genes	Function	Results and conclusion	Reference
Enamelin (ENAM) rs12640848	Enamel formation gene. Mineralization and structural organization of the enamel [16].	Analysis based on differences in allele frequency showed a statistically significant association with dental erosion and the ENAM rs12640848 marker. The frequency of the G allele of ENAM rs12640848 was significantly higher in the erosion group than in the nonerosion group.	Sovik et al. [31]

Amelogenin (AMELX) rs946252	Enamel formation gene. Mineralization during tooth enamel development [16].	When erosion severity was considered, statistically significant differences in allele frequency were observed for the AMELX rs946252 marker, with the C allele suggesting a protective role. An over-representation of the TT genotype of the AMELX marker was seen in cases with severe erosion. AMELX was also associated with severe erosion when the recessive model was considered; the TT genotype was significantly more frequent in the affected group than in the unaffected group. Association with severe dental erosion and the AMELX rs946252	Sovik et al. [31]
		When analyzing the terciles, significant associations were found between enamel loss and the AMELX rs946252. When comparing allele and genotype distributions between individuals more and less susceptible to enamel loss, no statistically significant differences were found.	Uhlen et al. [28]

Ameloblastin (AMBN) rs4694075	Enamel formation gene. Involved in enamel matrix formation and mineralization [16].	No evidence of association	Sovik et al. [31]

Tuftelin 1 (TUFT1) rs7526319	Enamel formation gene. Expressed in the developing and mineralized tooth [16].	No evidence of association	Sovik et al. [31]

Tuftelin 1 (TUFT1) rs4970957	Enamel formation gene. Expressed in the developing and mineralized tooth [16].	When analyzing the terciles, significant associations were found between enamel loss and the TUFT1 rs4970957. When comparing allele and genotype distributions between individuals more and less susceptible to enamel loss, no statistically significant differences were found.	Uhlen et al. [28]

Tuftelin-interacting protein 11 (TFIP11) rs5997096	Enamel formation gene. Thought to interact with tuftelin and can play a role in spliceosome disassembly in Cajal bodies [32].	No evidence of association	Sovik et al. [31]

Tuftelin-interacting protein 11 (TFIP11) rs134136 and rs5997096	Enamel formation gene. Thought to interact with tuftelin and can play a role in spliceosome disassembly in Cajal bodies [32].	When analyzing the terciles, significant associations were found between enamel loss and the both TFIP11 SNPs rs134136 and rs5997096. When comparing allele and genotype distributions between individuals more and less susceptible to enamel loss, no statistically significant differences were found.	Uhlen et al. [28]

SNP rs11681214 (located between peroxidasin (PXDN) and myelin transcription factor 1 like (MYT1L)	PXDN is involved in extracellular matrix formation and encodes a proteolytic enzyme, peroxidase, which is the main salivary antioxidant and found in the organic matrix of the enamel pellicle [33]	Statistically significant association between dental erosion and PXDN and MYT1L	Alaraudanjoki et al. [34]

(IRX1, IRX4) rs493321	Clustered near genes already proposed to be involved in embryogenesis or tooth development	Suggestively significant association between tooth wear and IRX1, IRX4 rs493321	Alaraudanjoki et al. [34]

Chromosome 8 open reading frame 86, fibroblast growth factor receptor 1 (C9orf86, FGFR1) rs11993596, rs112007639, rs12546327, and rs2461333	C8orf86's function is unknown. FGFR1 has been proposed to be the major receptor in the regulation mechanisms of fibroblast growth factor signals in human tooth development [35] and is involved in molar-incisorl hypoplasia [36]. FGFR1 also participates in the regulation of differentiation and secretory functions of odontoblasts and ameloblasts [37], and its role has been found to be critical in the formation of proper enamel [38].	Significant association between dental wear and C9orf86, FGFR1 rs11993596, rs112007639, rs12546327, and rs2461333 among males	Alaraudanjoki et al. [34]

(PRMT8) rs768398	Clustered near genes already proposed to be involved in embryogenesis or tooth development	Suggestively significant association between dental wear and PRMT8 rs768398	Alaraudanjoki et al. [34]

Cadherin 4 (CDH4) rs2426986, rs16984837, and rs6101273	The encoded protein (R-cadherin) is a calcium-dependent cell-cell adhesion glycoprotein, which is highly expressed in oral mucosa and also linked to both Wnt and FGF signaling [39]. Recently, it has been shown that CDH4 also promotes the expression of E-cadherin [40], which plays a crucial role in enamel development [41].	Statistically significant association between dental wear and CDH4 rs2426986, rs16984837, and rs6101273 among males	Alaraudanjoki et al. [34]

*γ*-protocadherin (PCDHG@) chr5: 141405828)	PCDHG@ is believed to modulate regulatory pathways critical for development, such as Wnt signaling (63), which is also critical at all stages of tooth development [34]	Evidence of a significant association in the GWAS on men, and the associations were also suggestively significant in the whole-sample GWAS	Alaraudanjoki et al. [34]

Fibroblast growth factor 1 (FGF1) and sprouty RTK signaling antagonist 4 (SPRY4) rs66756037	FGF1 and SPRY4 are involved in tooth development [42, 43]	The results demonstrate that there are loci in the genome that are directly associated with erosive wear. There is a difference between women and men in the genes associated with erosive tooth wear.	Alaraudanjoki et al. [34]

Stearoyl-CoA desaturase 5 (SCD5) chr4: 82684912	SCD5 has a role in activating the noncanonical Wnt pathway [44] as well as impairing cathepsin B secretion [45]. Cathepsin B is expressed during the maturation stage of the enamel [46] and also apparently has a role in dentine degradation [47].	Statistically significant association between dental wear and SCD5 chr4: 82684912 among females. In addition, 19 suggestive SNPs were identified near chr4: 82684912. Interestingly, this polymorphism is located in the chromosomal region 4q21 in which the genetic variation seems to impact dental caries experience and near locus 4q22, which showed the strongest suggestive association signal in the dental caries GWAS and harbors several tooth-related genes	Alaraudanjoki et al. [34]
Coagulation factor II thrombin receptor (F2R), F2R-like trypsin receptor 1 (F2RL1) chr5: 76795786	F2R, encoding protease-activated receptor 1 (PAR1), has recently been suggested to be highly expressed in secretory stage ameloblasts [48] and thus may have a critical role in the formation of proper enamel. This locus was also suggestive in the whole-sample GWAS and has been studied further in a fine-mapping study concerning dental caries [49].	Statistically significant association between dental wear and F2R, F2RL1 chr5: 76795786 among females	Alaraudanjoki et al. [34]

Msh homeobox 1 (MSX1) chr4	MSX1 is important for the tooth development [50]	A cluster of suggestive SNPs found near this gene when using the extreme opposite approach	Alaraudanjoki et al. [34]

Aquaporin 2 (AQP2) chr12: rs2878771	Potential involvement in immune response and salivary contribution [51]	Association found for genotypic (*p*=0.02) and dominant (*p*=0.03) models using logistic regression analysis, with the assumption that the dental erosive wear is a gene-environment complex model	Tulek et al. [2020] [52]

Aquaporin 5 (AQP5) chr12: rs3739306	Potential involvement in immune response and salivary contribution [53]	Association found for allelic model (*p*=0.02) using logistic regression analysis, with the assumption that the dental erosive wear is a gene-environment complex model	Tulek et al. [52]

**Table 2:** Candidate genes studied for dental caries in humans.

Genes	Function	Results and conclusion	Reference
Enamelin (ENAM)	Mineralization and structural organization of the enamel	Associated with higher caries experience	Patir et al. [54]; Shimizu et al. [49]; Jeremias et al. [55]; Gerreth at al. [56]; Wang et al. [57]
Amelogenin (AMELX)	Mineralization during tooth enamel development	Associated with higher caries experience	Deeley et al. [58]; Kang et al. [53]; Shimizu et al. [49]; Jeremias et al. [55]
		Associated with lower caries experience	Kang et al. [53]
		No evidence of association	Slayton et al. [59]; Olszowski et al. [60]; Ergoz et al. [61]; Gasse et al. [62]
Ameloblastin (AMBN)	Involved in enamel matrix formation and mineralization	Associated with higher caries experience	Patir et al. [54]; Shimizu et al. [49]; Ergoz et al. [61]
		Protective effect for caries	Gerreth at al. [63]
		No evidence of association	Slayton et al. [59]; Deeley et al. [58]; Jeremias et al. [55]
Tuftelin 1 (TUFT1)	Expressed in the developing and mineralized tooth	Associated with higher caries experience. Depends of the presence of *Streptococcus mutans*	Slayton et al. [59]; Patir et al. [54]; Deelay et al. [58]; Shimizu et al. [49]
		No evidence of association	Wang et al. [19]; Ergoz et al. [61]; Jeremias et al. [55]
Tuftelin interaction protein 11 (TFIP11)	Thought to interact with tuftelin and can play a role in spliceosome disassembly in Cajal bodies [32]	Associated with initiation of carious lesions and higher caries experience	Shimizu et al. [49]; Jeremias et al. [55]
Kallikrein-related peptidase 4 (KLK 4)	Degrades amelogenin	Associated with lower caries experiences	Wang et al. [19]; Abbasoglu et al. [64]; Gerreth at al. [63]; Cavallari et al. [65]; Weber et al. [66]
		No evidence of association when DMFT scores are analyzed	Slayton et al. [59]; Deeley et al. [58]; Patir et al. [54]; Shimizu et al. [49]; Ergoz et al. [61]; Yildiz et al. [67]
Matrix metalloproteinase 20 (MMP20)	Degrades amelogenin	Associated with higher caries experiences with poor oral health	Tannure et al. [68]
		No evidence of association	Wang et al. [19]
Matrix metalloproteinase 16 (MMP16)	Degrades amelogenin	Involved in white spot lesion and early childhood caries development	Antunes et al. [69]
		Significantly associated with caries in an individual sample of white adults	Lewis et al. [70]
CD14 molecule (CD14)	Mediates innate immune response to bacterial lipopolysaccharide	Absent in the saliva of individuals with active carious lesions	Bergandi et al. [71]
Human leukocyte antigen; major histocompatibility complex, class II, DR beta 1 (HLA-DRB1), and DQ beta 1 (HLA-DQB1)	Presents peptides derived from extracellular proteins	Frequency of allele 4 of DRB1 is increased in children with early childhood caries. Also, allele 2 of DQB1 is increased in adolescents affected by caries. DRB1 allele 1 and DQB1 allele 3 frequencies are increased in the presence of *Streptococcus mutans.*	Lehner et al. [72]; Altun et al. [73]; Bagherian et al. [74]; Valarini et al. [75]
Beta defensin 1 (DEFB1)	Antimicrobial peptide implicated in the resistance of epithelial surface to microbial colonization	Distinct DEFB1 haplotypes are associated with low and high caries experience	Ozturk et al. [76]; Krasone et al. [77]
Lactotransferrin (LTF)	Major iron-binding protein in milk and body secretions with antimicrobial activity	Associated with lower caries experience. No mutations found in the promoter region.	Azevedo et al. [78]; Fine et al. [79]; Brancher et al. [80]
Mucin 7 (MUC7)	Facilitates the clearance of bacteria in the oral cavity	Associated with higher caries experience with poor oral hygiene. No evidence of association.	Pol [81]; Buczkowska-Radlinska et al. [82]
Mucin 5B (MUC5B)	Contribute to the lubricating and viscoelastic properties of whole saliva	Suggested association with dental caries	Cavallari et al. [83]
Mannose-binding lection (protein C) 2, soluble (MBL2)	Bactericidal factor that binds to the Ra and R2 polysaccharides expressed by certain enterobacteria	No evidence of association	Olszowski et al. [60]
Aquaporin 5 (AQP5)	Water channel protein that plays a role in the generation of saliva, tears, and pulmonary secretions	Associated with higher caries experience	Wang et al. [19] Weber et al. [66]
		Possibly protective for caries in interaction with fluoride	Anjomshoaa et al. [84]
Protein-rich protein HaeIII subfamily 1 (PRH1)	Provide protective environment for the teeth	Associated with higher caries experience and colonization by *Streptococcus mutans*	Zakhary et al. [85]
Matrix metalloproteinase 2 (MMP2)	Degrades type IV collagen	No evidence of association	Tannure et al. [86]
Matrix metalloproteinase 9 (MMP9)	Degrades type IV and V collagens	No evidence of association	Tannure et al. [86]
Matrix metalloproteinase 13 (MMP13)	Involved in endochondral ossification and bone remodeling. This gene is physically close to MMP20 and may be evolved at the same time before the divergence of ray-finned fish and lobe-finned fish [87]	Associated with lower caries experience	Tannure et al. [86]
TIMP metallopeptidase inhibitor 2 (TIMP2)	Possibly critical to the maintenance of tissue homeostasis by suppressing the proliferation of quiescent tissues in response to angiogenic factors and by inhibiting protease activity in tissues undergoing remodeling of the extracellular matrix	No evidence of association	Tannure et al. [86]
Dentin sialophosphoprotein (DSPP)	Involved in the mineralization process of dentin	Associated with lower caries experience	Wang et al. [19]
Secreted phosphoprotein 1 (SPP1)	Involved in the attachment of osteoclasts to the mineralized bone matrix	No evidence of association	Wang et al. [19]
Arachidonate 15-lipoxygenase (ALOX15)	Associated with bone mineralization, possibly involved in the formation of the hard structures of teeth	Association with early childhood caries	Abbasoglu et al. [64]
Carbonic anhydrase IV (CA4)	Involved in respiration, calcification, acid-base balance, bone resorption, and formation of aqueous humor, cerebrospinal fluid, saliva, and gastric acid	No evidence of association	Yarat et al. [88]
Carbonic anhydrase VI (CA6)	Catalyzation of the reversible hydration of carbon dioxide in saliva, possible involvement in pH regulation, taste perception, and tooth formation	Associated with *S. mutans* colonization, tooth biofilm microbiota composition, and risk of dental caries	Esberg et al. [89]
Distal-less homeobox 3 (DLX3)	One of the genes involved in amelogenesis imperfecta	Associated with dental caries susceptibility	Ohta et al. [90]
Taste receptor, type 2, member 38 (TAS2R38)	Controls the ability to taste glucosinolates	Associated with lower caries experience	Wendell et al. [91]
Taste receptor, type 1, member 2 (TAS1R2)	Sweet taste receptor	Associated with both lower and higher caries experience	Wendell et al. [91]; Kulkarni et al. [92]
Guanine nucleotide binding protein, alpha transducing 3 (GNAT3)	Believe to be involved in dietary preferences	No evidence of association	Wendell et al. [91]
Solute carrier family 2 (facilitated glucose transporter), member 2 (SLC2A2)	Mediated facilitated bidirectional glucose transport	Associated with higher caries experience	Kulkarni et al. [92]

## Data Availability

The data were collected from the National Library of Medicine database.
